# Emergent Runaway into an Avoidance Area in a Swarm of Soldier Crabs

**DOI:** 10.1371/journal.pone.0097870

**Published:** 2014-05-19

**Authors:** Hisashi Murakami, Takenori Tomaru, Yuta Nishiyama, Toru Moriyama, Takayuki Niizato, Yukio-Pegio Gunji

**Affiliations:** 1 Department of Earth & Planetary Sciences, Kobe University, Kobe, Hyogo, Japan; 2 Science & Technology Entrepreneurship Laboratory (e-square), Osaka University, Osaka, Japan; 3 Faculty of Textile Science and Technology, Shinshu University, Ueda, Nagano, Japan; 4 Faculty of Engineering, Information and Systems, Tsukuba University, Tsukuba, Ibaraki, Japan; 5 Department of Intermedia Art and Science, School of Fundamental Science and Engineering, Waseda University, Tokyo, Japan; Keio University, Japan

## Abstract

Emergent behavior that arises from a mass effect is one of the most striking aspects of collective animal groups. Investigating such behavior would be important in order to understand how individuals interact with their neighbors. Although there are many experiments that have used collective animals to investigate social learning or conflict between individuals and society such as that between a fish and a school, reports on mass effects are rare. In this study, we show that a swarm of soldier crabs could spontaneously enter a water pool, which are usually avoided, by forming densely populated part of a swarm at the edge of the water pool. Moreover, we show that the observed behavior can be explained by the model of collective behavior based on inherent noise that is individuals’ different velocities in a directed group. Our results suggest that inherent noise, which is widely seen in collective animals, can contribute to formation and/or maintenance of a swarm and that the dense swarm can enter the pool by means of enhanced inherent noise.

## Introduction

Emergent behavior that brings about a mass effect is one of the most striking aspects of collective animal groups. In recent years, developments in image analysis have made it possible to obtain kinetic data on the movements of real organisms [1]–[5] and to compare that data with simulation models [6], [7]. In contrast, there are few comparisons between these models and data obtained using behavioral experiments [8], [9]. Rather, many behavioral experiments that have used animal groups were conducted in the context of social learning and/or the opposed relationship between an individual and society, such as that between a fish and a school [10]–[12]. Investigating such a behavior, however, must help to find new mechanisms underlying interactions between individuals.

Berdahl and colleagues [8] used schooling fish, taking advantage of the avoidance of light areas (or preference for darker area such as the habitat under the lee of a rock) as one approach to the problem of linking experimental behavior results to modeled behavior. In this experiment, a temporally changing light field that controlled the mean light level was projected onto a tank in which the fish were swimming. They found that if the size of the school became larger, the level of performance in a dark area increased. In short, collective sensing against light areas was enhanced in larger schools. Moreover, by comparing this with the model, they concluded that this situation arose from the rule of attraction that has been proposed in some theoretical models such as BOIDS [13] and the zone-based model [14] by which an agent approaches neighbors if they are separated from each other. This raises the question of cases of spontaneously invading an avoidance area, which are frequently found in biology. [15], [16] In this case, it would be expected that a different rule from the attractive explanation is required.

In this study, we conducted an experiment with respect to invading avoidance areas using a swarm of soldier crabs, *Mictyris guinotae* [17]–[20], which live in the tideland and can form large swarms. Through numerous field surveys of soldier crabs, we found the following observations to be characteristic of soldier crab general swarming behavior: (i) A swarm moving in the tideland has inherent noise. In other words, crabs have different velocities in maintaining a directed swarm, which reveals the local turbulent flow in a swarm. (ii) When a swarm faces a pool that has been naturally generated on a tideland, it does not enter this avoidance area if the swarm is small or sparse. In contrast, if the swarm becomes bigger and forms a dense region, this part of the swarm rushes into the pool without pausing. In other words, turbulent motion results in part of the swarm becoming highly concentrated, and this part enters and crosses the water due to the effect of the group. (iii) Individuals in other parts of the swarm follow their predecessors.

Based on these observations, in particular observation (ii), we designed an experiment for the water crossing behavior of soldier crabs, *M. guinotae*. To investigate the behavior, we created an apparatus with a water pool sandwiched between two shore areas under semi-natural conditions and made comparisons between small (5 individuals) and large (15 individuals) swarms with respect to the performance of water crossing behavior. Then, we estimated whether the performance changed depending on the size of the swarm concentrated at the edge of the water pool. Finally we compared the experimental results with a swarm model based on inherent noise that was proposed in our previous study [21]–[24].

## Materials and Methods

### Soldier Crabs *Mictyris guinotae*

We studied *M. guinotae* living in Funaura Bay on Iriomote Island, Okinawa Prefecture, Japan. Soldier crabs, whose carapace sizes are approximately 15 mm, are among the few crabs adapted to walking forwards, rather than sideways [17]–[20]. Although they burrow tunnels and live under substrate at higher tidal levels, they emerge and feed on detritus on the lagoon surface in swarms at the lower tidal level. During the breeding season (from December to March), their behavior differs depending on mating [25]. To avoid these effects, our experiment was conducted in the daytime during the 4 hours around ebb tide on fine weather days in October 2013. Crabs were collected in plastic containers that contained mud substrate and were separated into each swarm size in the 5 minutes before each trial. For each trial different swarm composed of different crabs were used without pre-training, with a total of nine hundred crabs used throughout the experiment. Crabs were immediately released after the experiment. No specific permits were required for the described field studies and the locations are not privately-owned or protected in any way. *M. guinotae* is not endangered or protected species.

### Experimental Setup

**Figure 1:**
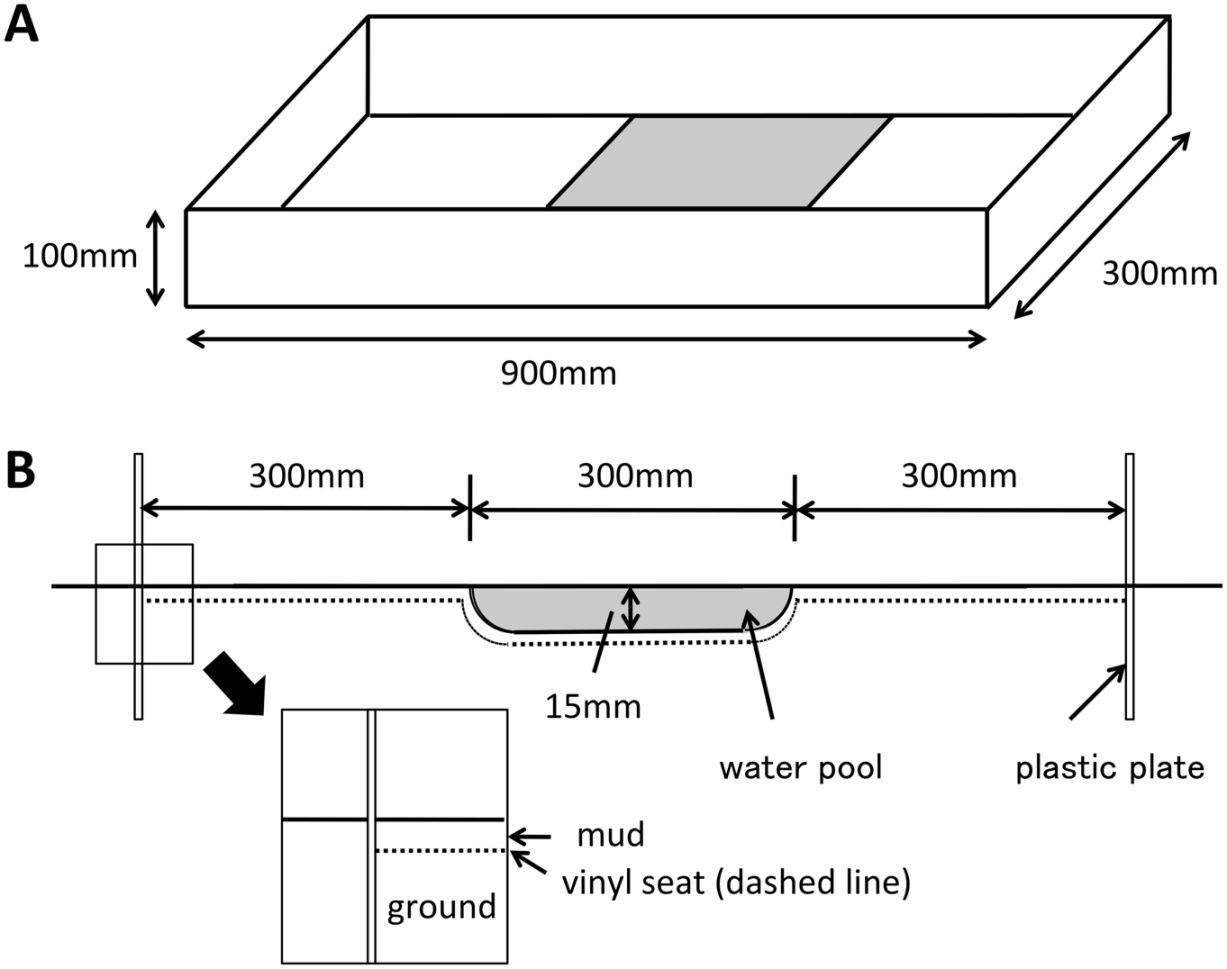
Experimental apparatus for water crossing behavior. (A) Plastic plates surround the tidal land to form a rectangular shape. The pale grey areas represent the water pool. (B) Cross section of apparatus. The water pool is sandwiched between two shore areas.

A simple apparatus was constructed on the tideland (figure 1). We formed a rectangle by inserting plastic plates vertically, 100 mm deep into a flat area on the tideland (300×900 mm, 100 mm height). To make a pool (300×300 mm, 15 mm depth) sandwiched between two shore areas, we dug out the central part of the tideland surrounded by the plates and covered the rectangular area with a vinyl sheet onto which some mud was added. The pool was then filled with water obtained from a naturally generated pool. The vinyl sheet was used to inhibit crabs from burrowing tunnels and to keep the water in the pool. The crabs did not burrow tunnels during any trials and the water level changed little. Before each trial, we leveled the shore areas and resupplied water to the pool to maintain conditions.

Each swarm was gently thrown onto one side of the shore area apparatus, and swarm behavior was recorded for three minutes with a video camera (Panasonic HDC-TM700, 1920×1080 pixels). We used image-processing software (ImageJ; Rasband, W.S., National Institutes of Health, Bethesda, Maryland, USA) to calculate inter-individual distances (at 3 seconds intervals). We obtained x–y coordinates for each crab as a single pixel whose side length was 0.56 mm.

### Swarm Model

Here we describe our swarm model in detail. We present a rough idea of the model in the results section.

Firstly, we describe basic behavior of the model.

Our swarm model consists of *N* individuals moving in discrete time and in a discrete 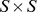 space where 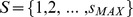. The location of the *k*-th individual at the *t*-th step is given by
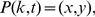
(1)where 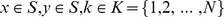, and the boundary condition is given as wrapped fashion. Each *k*-th individual at the *t*-th step has *P* number of potential vectors 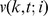 with 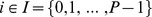. If 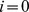, the vector 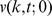, called the principal vector, is represented by the angle *θ_k,t_*, such that
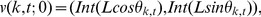
(2)where for any real number *x*, *Int*(*x*) represents integer *X* such that 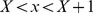. *L* is the length of principal vector. Because of the wrapped fashioned boundary condition, 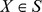. If 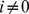, the vector is defined using a random value, *η,* selected with equal probability from [0.0, 1.0] and a random value (radian), *ξ*, selected with equal probability from [-*απ, απ*], as
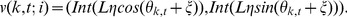
(3)


The principal vector 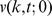 is a special case where 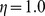 and 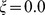. For each 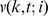, the target of the vector is represented by 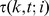 such that



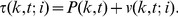
(4)


To implement mutual anticipation, we define the popularity of the targets of the vectors. The popularity is defined for each site at the *t*-th step, (*x, y*) with 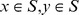, by
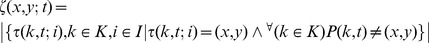
(5)


Before updating the location, for any (*x, y*) at the *t*-th step, we set
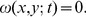
(6)


Updating the location of individuals is asynchronously executed. The order of updating is randomly determined independent of the number of individuals, *k*. If there exists 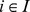 such that
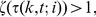
(7)the next site for the *k*-th individual is defined by
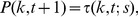
(8)where *s* satisfies the condition such that for any 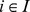,



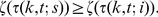
(9)


In other words, an individual moves to the target of its own potential vector that has maximum popularity. If there multiple sites satisfy condition (9), one of them is chosen randomly.

Because updating is asynchronous, a set of sites updated by equation (8) is gradually grows. A set of updated sites is represented by 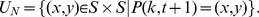


An individual that satisfies condition (7) and moves by equation (8) is called a wanderer. The vacated site generated by a moving wanderer is recorded in memory by
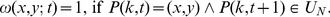
(10)


After all wanderers have been updated, an individual which does not satisfy condition (7) moves to the vacated site in follower-neighborhood, *N_f_*, by
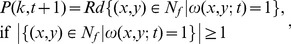
(11)where *RdJ* represents an element randomly chosen from set *J*. An individual whose movement is determined by equation (11) is called a follower.

If an individual is neither wanderer nor a follower, it moves by
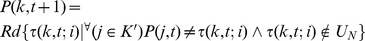
(12)where *K’* is an index set of individuals that are not updated.

Finally, principal vectors are locally matched with each other in the neighborhood through velocity matching, *M*. This matching operation is expressed as
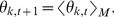
(13)


The bracket with *M* represents the average velocity direction in the neighborhood, *M*. The parameters in our model are listed below.

*L*: the length of principal vector

*P*: number of potential vectors

*α*: angle derived from the principal vector

*R_f_*: radii of the follower-neighborhood

*R_M_*: radii of the neighborhood of velocity matching

Secondly, we define water crossing behavior in our model

In the simulation introducing the pool, we define a specific area 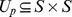 in which the condition (7) that allows mutual anticipation (equation (8)) is replaced by
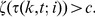
(14)


In the simulation, we set at 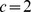. Hence, it is more difficult for individuals to go through the area *U_p_*, which mimics a water pool that an individual soldier crab does not enter. Only by introducing the specific area *U_p_*, can we simulate the water crossing behavior.

Finally, we show tendency to walk along a wall in our model.

We first define the wall state for any lattice (*x, y*) such that
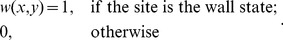
(15)


In the simulation of water crossing behavior, an agent can be located only at a site where 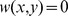. The angle of tangential direction is defined for each wall state site (*x, y*) and is represented by *θ_w_*(*x, y*). The tendency of walking along a wall is defined by
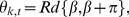
(16)

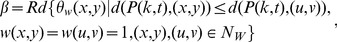
(17)where *d*((*p, q*), (*x, y*)) represents the metric distance between two sites (*p, q*) and (*x, y*), and *N_W_* represents the neighborhood of wall-monitoring for each agent with a radii 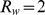. If an agent is close to the wall with respect to *N_W_*, the agent’s velocity, *θ_k,t_* is parallel to the tangential direction of the wall. After this operation, velocity matching (equation (13)) is applied to all agents. Only from (16) and (17) can agents close to the wall walk along the wall.

## Results

### Experimental Results

To investigate whether the water crossing behavior was caused by mass effect, we compared small swarms (5 individuals) with large swarms (15 individuals) with respect to the performance of water crossing behavior. Each trial was conducted for three minutes and a total of 40 trials were performed for each swarm size. For each trial, the success rate *φ* was defined by the number of individuals that completely walked across the water pool, normalized by the total number of individuals. The success rate *φ* was zero if no crabs completely walked across the pool, while it was one if all of the crabs finished crossing to the opposite shore. According to field observations of naturally generated water crossing behavior, there were some crabs left in the water pool, so not all individuals composing the swarm finished crossing to the other shore. Hence, we defined a success trial by *φ*>0.5, a failure by *φ*≤0.5, and the performance of water crossing behavior by the number of success trials normalized by the total number of trials.

**Figure 2:**
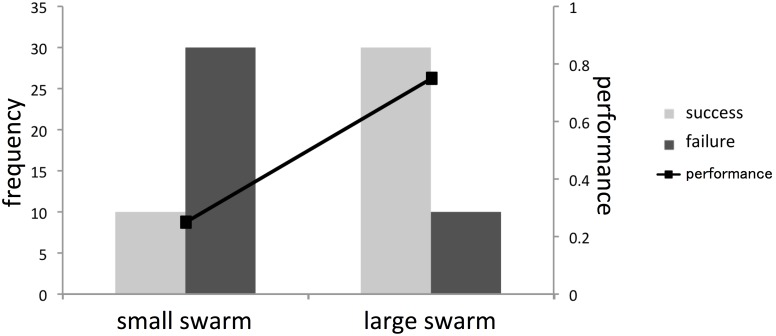
Frequency of success and failure as well as performance of each water crossing behavior. Large swarm, *N* = 40; small swarm, *N* = 40.

Figure 2 shows the performance of water crossing behavior and the frequency of successful and unsuccessful trials. The performance differed significantly between small and large swarms (performance: 30/40 (large swarm) vs. 10/40 (small swarm), Fisher exact test: P<0.001). The number of failed trials performed by small swarms was significantly larger than the number of successful trials (failure trial: 30 out of 40 swarms, binomial test: P<0.01). The number of successful trials performed by large swarms was significantly larger than the number of failures (success trial: 30 out of 40 swarms, binomial test: P<0.01). These results indicate that while small swarms avoid entering the water, large swarms cross over the water pool. This can be interpreted as emergent behavior caused by a mass effect.

Detailed results of the above analysis that show the success rate *φ* and *NI* of each trial are provided in Table 1. *NI* indicates the number of individuals that eventually crossed the pool during a trial. Between small and large swarms, we found a significant difference in the number of trials in which no crab crossed the river (0/40 (large swarm) vs. 14/40 (small swarm), Fisher exact test: P<0.001). This result shows that small swarms avoid the water. There is, however, no significant difference in the number of trials in which all crabs finish crossing to the opposite shore (7/40 (large swarm) vs. 2/40 (small swarm), Fisher exact test: P>0.05, NS), which means that crabs composing the swarm do not always finish crossing, independent of swarm size, as seen in natural conditions.

**Table 1:** Detailed results for the analysis shown in figure 2.

Small swarms						Large swarms					
Trial	*φ*	*NI*	Trial	*φ*	*NI*	Trial	*φ*	*NI*	Trial	*φ*	*NI*
1	0	**0**	21	0.2	1	1	0.6	9	21	0.73	11
2	0.8	4	22	0	**0**	2	0.87	13	22	0.87	13
3	1	**5**	23	0	**0**	3	0.67	10	23	0.93	14
4	0.2	1	24	0.6	3	4	0.67	10	24	0.67	10
5	0.6	3	25	0.4	2	5	0.87	13	25	0.87	13
6	0.8	4	26	0.2	1	6	1	**15**	26	0.27	4
7	0.6	3	27	0.2	1	7	1	**15**	27	0.33	5
8	0.8	4	28	0.4	2	8	1	**15**	28	0.2	3
9	0.2	1	29	0.4	2	9	1	**15**	29	1	**15**
10	0	**0**	30	0	**0**	10	0.93	14	30	0.93	14
11	0	**0**	31	0.2	1	11	1	**15**	31	0.73	11
12	0	**0**	32	0.6	3	12	0.2	3	32	0.73	11
13	0.2	1	33	0	**0**	13	1	**15**	33	0.67	10
14	0.2	1	34	0	**0**	14	0.73	11	34	0.07	1
15	0.4	2	35	0	**0**	15	0.6	9	35	0.4	6
16	0.2	1	36	0	**0**	16	0.6	9	36	0.27	4
17	0	**0**	37	0.4	2	17	0.67	10	37	0.73	11
18	0.2	1	38	0	**0**	18	0.8	12	38	0.4	6
19	0	**0**	39	0.2	1	19	0.47	7	39	0.87	13
20	1	**5**	40	0.8	4	20	0.33	5	40	0.87	13

The success rate *φ* was defined by the number of individuals that completely walked across the water pool during each trial, normalized by the total number of individuals. *NI* indicates the number of individuals that eventually crossed the pool during each trial.

To estimate the contribution of the density effect on the water crossing behavior in detail, we investigated whether the performance depended on the size of the swarm concentrated at the edge of the water pool. First, to equalize the conditions when the swarm entered the water, we defined the initial condition such that there was no crab in either the pool or on the opposite shore area at a certain time step and that in the next time step a crab entered the pool. Note that we only used crab positional data at each of the time steps, which were separated by three seconds intervals, and ignored crab behavior between time steps. Second, to calculate the size of the swarm concentrated at the edge of the pool, we defined the swarm network as a swarm that consisted of individuals within 50 mm of another individual; such individuals were connected to each other as the nodes of the network. We calculated the size of the swarm network that a crab entering into the pool belonged to and checked the performance for each swarm network. As long as it satisfied the initial condition, we continued to check the performance for each swarm network until each trial finished. In this analysis, we defined a failure trial as a trial in which all individuals comprising a network did not finish crossing the pool and a successful trial as a trial in which at least one individual within the network completely walked across the water pool. The performance of water crossing behavior was defined as the number of successful trials normalized to the total number of trials.

**Figure 3:**
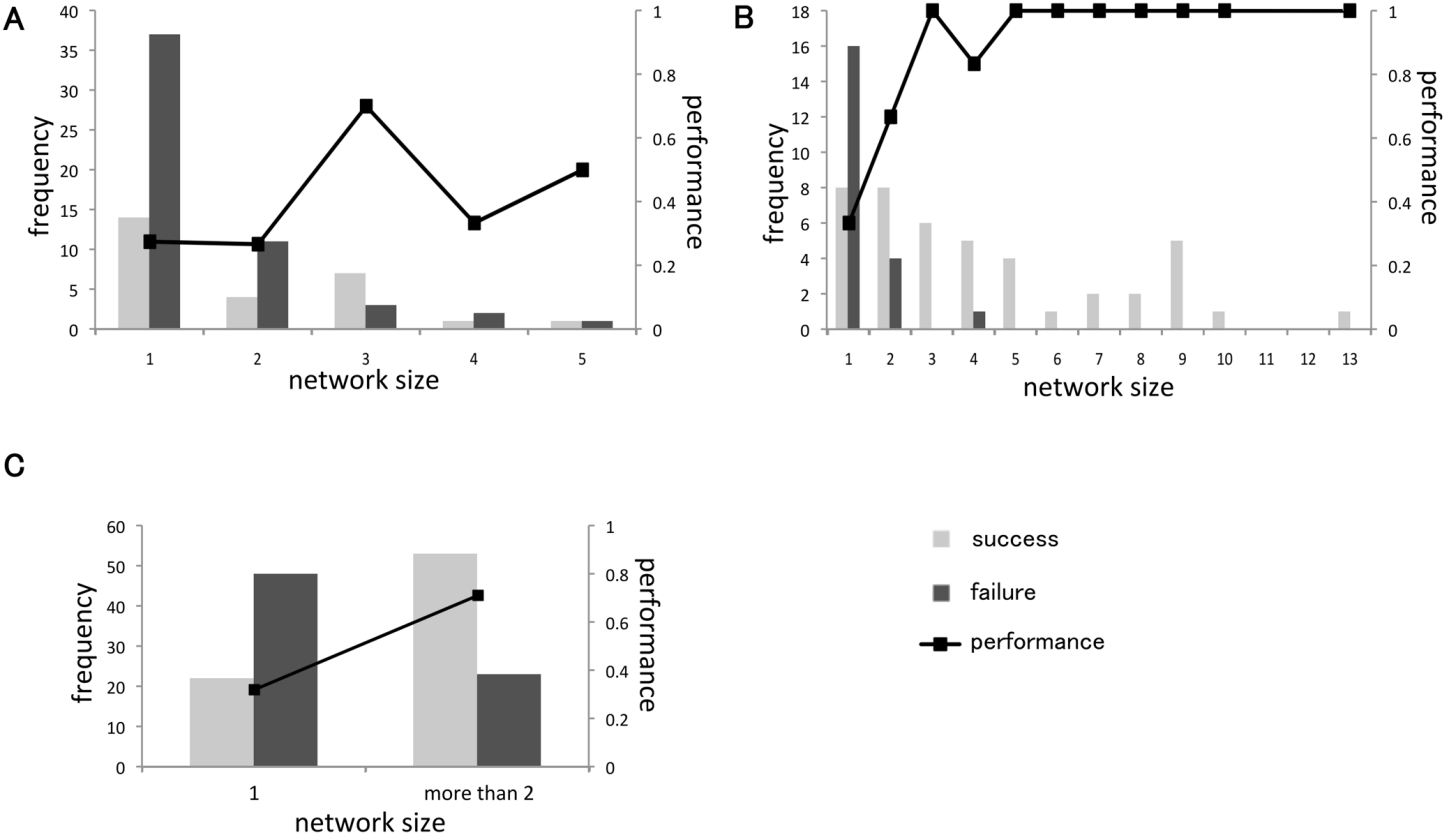
Frequency of success and failure of each network size as well as the performance. (A) Small swarm trials, *N*  = 81. (B) Large swarm trials, *N* = 65. (C) Solitary crabs trials (i.e., network size is one) (*N* = 70) and a swarm whose network size was more than two (*N* = 76).

Figure 3A and B show the performance of water crossing behavior and frequency of success as well as the failure of small and large swarms, respectively. It is easy to see that the smaller network size was (in particular when the network size was one), the smaller the performance was for both small and large swarms. When the network size was more than six, every swarm successfully crossed the water. When we compiled the data sets for both large swarms and small swarms, we found significant differences in the performance between solitary crabs (i.e., network size is one) and swarms with network sizes that were greater than two (performance; 53/76 (swarm) vs. 22/70 (solitary crab), Chi-square test: *χ* ^2^_1_ = 19.900, P<0.001) (figure 3C).

**Figure 4:**
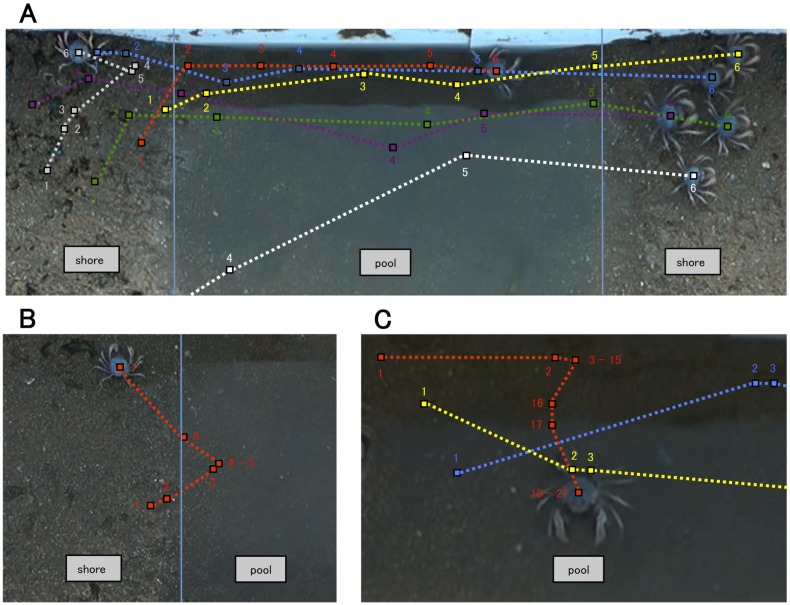
Typical snapshots of water crossing behavior with several trajectories. Different colors correspond to different crabs. Trajectories are composed of small squares that represent the location of each crab and dashed lines that connect its location at a certain time with its location at the next time. The number next to each square represents the order of time, with a time interval of three seconds. The shore area and pool area are divided by a blue vertical line. (A) Example of a successful water crossing. (B) Example of a failed behavior. (C) Example of a crab being left in the pool despite the crossing of the other crabs.

Figure 4 shows typical snapshots of the water crossing behaviors with some trajectories. Each crab is represented by a different color. Trajectories are composed of small squares representing each crab’s location and dashed lines that connect the locations at one time with those at the next time. The number next to each square represents the order of time, with a time interval of three seconds. The blue vertical line in figure 4A, B represents the border between the shore area and the pool area. Figure 4A provides examples of successful water crossings. It shows the effect of gathering at the edge of pool; once a swarm composed of six crabs entered into the pool, it crossed over the pool without stopping. Figure 4B provides an example of failure of the behavior. Although the crab entered the pool at least once, it hesitated in the water and left the pool. In addition, even though other crabs crossed over the pool, a crab was left in the pool, as was seen in natural conditions (figure 4C).

### Swarm Model based on Inherent Noise

Here, we show a swarm model to explain emergent water crossing behavior. As mentioned in the introduction section, using numerical field observations and experimental results we found the following characteristics of general swarming behavior in soldier crabs: (i) a swarm moving in a lagoon has inherent noise and maintains coherence; (ii) turbulent motion results in part of the swarm becoming highly concentrated, and this part enters and crosses the water through an effect of the group; and (iii) individuals in other parts of the swarm follow their predecessors. Characteristic (i) suggests perpetual negotiation among individuals with respect to direction. Characteristic (ii) reveals that density affects the mechanism to generate a swarm. Such an inherent noise has been found not only in the swarming of soldier crabs but also in other animal groups. For example, in a starling flock each bird continuously changes its neighbors and reveals supper-diffusive behavior in the center of the mass reference frame of the flock [4]. Moreover, it has been reported that a noise inherently generated within a locust-march plays an essential role in the collective change of direction [9]. When considering (i) combined with (ii), it is suggested that inherent noise positively contributes to generate and maintain a swarm.

**Figure 5:**
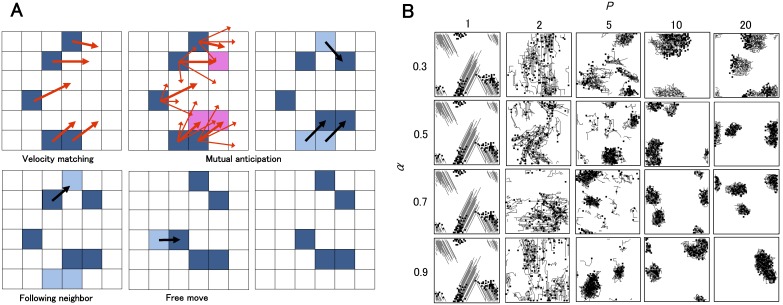
Schematic diagram of the model and the behavior of a model simulation. (A) Illustration of a transition of a swarm in lattice space. Each individual is represented by a blue cell and their potential transition is represented by a red arrow. The principal vector is represented by a thick red arrow. First, velocity matching is applied to the principal vectors (upper left). Next, the mutual anticipation is estimated. The amount of overlap between targets of potential transitions is calculated and sites with an overlap larger than 1 (threshold) are obtained (pink site). For example, the popularity of the highest pink site is 2 (upper center). An individual whose potential transitions reach some popular sites moves to the most popular site (black arrow in the upper right). After that, followers (lower left) and free wanderers (lower center) move, which results in the final distribution (lower right). (B) How changing *P* and *α* affects the patterns of a swarm composed of 100 individuals in a 50×50 lattice. Each individual is represented by a square and a trajectory tail, where *L* = 4, *R_f_* = 2, and *R_M_* = 3.

To incorporate these soldier crab swarm behaviors into a model, we introduced several potential transitions for each individual that allowed the individual to anticipate the movements of other individuals within the swarm (figure 5A). Each individual has its own principal vector (velocity) accompanied by a number of potential transitions (*P*) in a range restricted by the angle (*α*) and the length (*L*). If the targets of any potential transitions overlap at a certain site (lattice), the number of potential transitions to that site is counted as the site’s “popularity”. If there are multiple sites with a popularity larger than 1 (threshold value) among an individual’s potential transitions, it is assumed that the individual moves to the site with the highest popularity. If several individuals intend to move to the same site, one individual is randomly chosen to move there and the others move to their second most probable site. This rule represents the mutual anticipation of the individuals. For example, people often manage to avoid collisions and walk in a crowd of others using anticipation [26], [27]. Therefore, we implement this type of behavior into our model. If there is no site with a popularity exceeding the threshold value in the neighborhood of an individual, and if others within a radius *R_f_* move due to mutual anticipation, the individual moves to occupy the absent site generated by mutual anticipation. Namely, it follows its predecessor. If an individual’s movements are not based on mutual anticipation or the actions of a predecessor, it moves in the direction of a randomly chosen potential transition. This type of individual is called a “free wanderer” (see further details in Materials and Methods).

We implemented these rule in an asynchronous updating model in a lattice space coupled with velocity matching (VM) of principal vectors (figure 5A). VM is implemented in a neighborhood with a radius *R_M_*. By assuming a maximum of one individual per lattice, the model implements collision avoidance (CA). The predecessor-following rule is also an example of flock centering (FC) (also known as attractive rule). The rules of VM, CA, and FC constitute BOIDS. BOIDS has recently been expanded to model more realistic characteristics of flocks and schools [14], [28]–[31]. Thus, the introduction of mutual anticipation to our model is a natural extension of BOIDS. Figure 5B demonstrates how various swarming patterns in the model with rapped boundary conditions depend on the parameters *α* and *P* with *R_f_* = 2 and *R_M_* = 3. If *P* is 1, the model mimics BOIDS. If *P* is 2, multiple potential transitions break out in collective motion because two transitions contribute not to make a popular site, but to make a random transition. If *P* exceeds 2, mutual anticipation contributes to swarm formation, especially if *α* is large. It is easy to see that a swarm contains turbulent motion despite maintaining a highly dense whole when *P* is larger. Furthermore, if there is a solitary individual, it is regarded as a free wanderer and moves by choosing randomly from potential transitions. Therefore, the larger *P* is, the more random an individual’s move is. In this sense, we can regard potential transitions as inherent noise. In our previous study, we showed that this model could explain several phenomena exhibited by animal groups [21]–[24].

### Water Crossing Behavior in the Swarm Model

In this section, we show the water crossing behavior performed by our swarm model, setting the parameters at *P* = 10, *α* = 0.5, *L* = 4, *R_f_*  = *R_M_* = 2. Emulating the experiment conducted with real swarms, we set the bounded space to consist of 90×30 lattices in which the pool area (30×30 lattices) was sandwiched between the shore areas (each 30×30 lattices). The pool, as an avoidance area, was defined as a specific area in which the condition allowing mutual anticipation of potential transitions were overlapped larger than two (see Materials and Methods). Hence it was more difficult for individuals to go through the specific area, which mimics a natural pool that an individual soldier crab does not enter. In this simulation, individuals could not go outside the bounded space, emulating the wall of the experimental apparatus, even though potential transitions overlapped with the area outside of the space. In addition, individuals were given the tendency to walk along the boundary of the space because it has been reported that real solder crabs tend to walk along the wall (see Materials and Methods).

**Figure 6:**
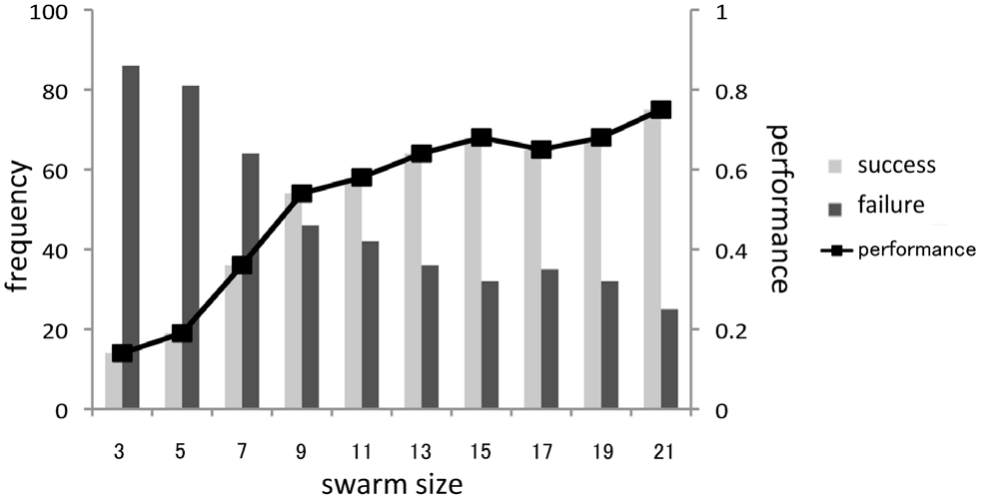
Frequency of success and failure as well as the performance of each swarm size. It is clear that if the swarm is bigger, the success rate of crossing the water increases (*N* = 100 for each swarm size).

In each simulation, individuals were randomly allocated to one of the shore areas with random directions of principal vectors. We conducted each trial for 250 time steps and ran 100 trials for swarms with 3, 5, …, 21 individuals. Successful trials, failures, and the performance of water crossing behavior were defined by using the success rate *φ* along with the experiment conducted with real crabs. Figure 6 shows the performance of water crossing behavior and the frequency of success and failure for each swarm size. It is easy to see that the bigger the swarm size, the more success in water crossing. When we compared the performance between swarms composed of five (small) and fifteen (large) individuals that were compared in the real soldier crab experiment, there were significant differences between these swarms (performance: 68/100 (fifteen individuals) vs. 19/100 (five individuals), Chi-square test: *χ* ^2^_1_ = 39.872, P<0.001).

In the experiment, because of difficulty in collecting a huge number of fresh crabs, we only tested two sizes of swarms (i.e., 5 and 15 individuals) with real crabs. However, we can predict the behavior of intermediary size of swarm by using our swarm model. In figure 6, it can be observed that the performance of water crossing behavior of our model exceeds 0.5 i.e., the number of successes is over the number of failures when the swarm size is more than nine. Hence it would be expected that if we test a swarm composed of over nine real crabs, the swarm success the water crossing behavior on more than half of trials.

**Figure 7:**
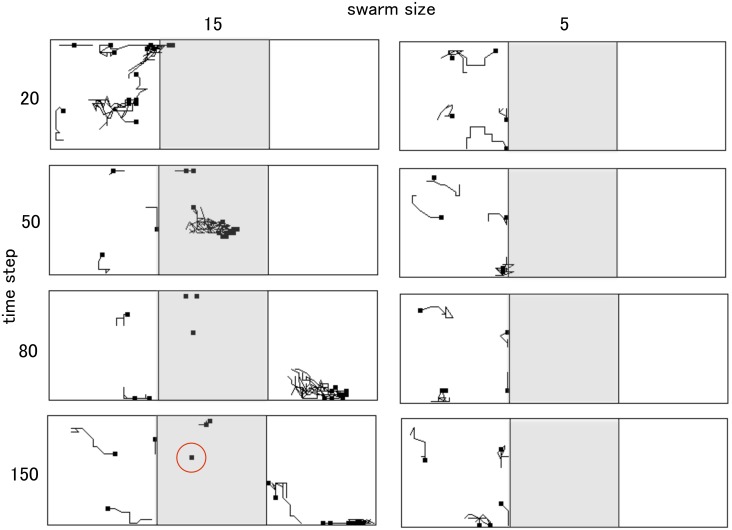
Snapshots of the time development of swarm trajectories in a model simulation. The pale grey area located in the center indicates the pool area. An individual left in the pool is indicated by a red circle.

Figure 7 shows snapshots of the time development of swarm trajectories in a model simulation. Each agent is represented with its 10-step trajectories. The pale grey area located in the center indicates the pool area in which the threshold to allow mutual anticipation is heightened. In the case of a swarm whose size is five, solitary or few individuals do not enter into the pool, even though the swarm walks along the marginal area of the pool. On the other hand, in the case of a swarm whose size is fifteen, dense swarm is formed at the edge of pool, and once individuals enter into the pool they cross over the pool without stopping. Moreover, we observed that some individuals remained in the pool despite the crossing over of the others, which is frequently observed with real soldier crabs (indicated by red circle in figure 7). Therefore, the simulated swarm appropriately mimics the behavior of real soldier crabs as shown in the experiment and natural conditions.

## Discussion

In this study, we first conducted an experiment with respect to water crossing behavior of soldier crab swarms. We observed that when a small swarm confronted a waterfront, it could not enter a water pool. Hence, when a swarm was small or sparse, they regarded the water pool as an area to avoid entering. In contrast, when the swarm became bigger and a highly concentrated part was created inside the swarm, they could then enter and cross the water. Therefore, by creating a large and dense region, the swarm could overcome the water pool as an avoidance area. Although spontaneous invading behaviors into an avoided area have been reported for several animals, in most cases, animals have some motivation such as rich food sources to encourage entrance into an avoidance area [15], [16]. Our experimental results indicate that the behavior of overcoming the avoidance area observed in soldier crab swarming is obviously an emergent behavior because, while small swarms passively responds to a water pool as a noxious stimulus, if the swarm becomes bigger they spontaneously enters to the pool. Some examples of emergent behavior of collective animals were presented previously [8], [32]. In particular, Berdahl and others [8] revealed that greater group-level responsiveness to the environment arises spontaneously as group size increases. For example, a fish school stays away from the light as an avoidance area more sensitively if it is bigger in size. They explain this emergent behavior by the simple rule of attraction such that an agent approaches its neighbors if they separate from each other.

The water crossing behavior can be explained by our model, which incorporated inherent noise and mutual anticipation. Inherent noise in the model created diverse potentials for each individual and the swarm collapsed if each individual chose potential transitions randomly. In contrast, if the diversity of moves is used for other individuals’ anticipation, this results in a densely collective motion. Mutual anticipation may be affected by each individual crab’s sensitivity to the moves of other individuals and by the asynchronous movements of the individuals in a swarm. This sensitivity allows crabs to detect the site to which many individuals could move toward. Because of asynchronous updating, crabs can move in various directions without collision and overcrowding.

By considering the water pool to be an area where it is difficult for mutual anticipation to occur, the water crossing behavior is easily simulated in our model. In simulations with small swarms, even if it is faced with the marginal area of the pool, it cannot enter the pool because potential transitions are not overlapped enough for mutual anticipation of the swarm to occur in the pool. For a large swarm, however, after regions of high concentration are formed, the swarm can enter the pool and make mutual anticipation at sites in the pool due to highly concentrated potential transitions. Then, even when an individual cannot occur due to mutual anticipation in the pool, it is not separated from swarm as long as it can follow the predecessor, and hence the swarm crosses over the pool without stopping. However, if the individual cannot use either mutual anticipation or follow its neighbors, it is separated from swarm and left in the pool, as was seen in the experiment. In this way, our swarm model can emulate the behavior of real soldier crabs to some extent. It is difficult to explain the behavior of entering an avoidance area only with the simple attraction rule because when a local part of a swarm enters into the water pool and the greater part of it stays on the shore around the pool, the local part is brought back to the greater part by following the attraction rule.

Inherent noise can be widely seen in animal groups [4], [9], [33], [34]. By using mutual anticipation, inherent noise that could be expected to negatively impact the swarm, makes a positive contribution to the collective motion and can explain the water crossing behavior of a soldier crab swarm. These results suggest that inherent noise and mutual anticipation play an important role in understanding collective behavior.
